# Magnetic field effect on the energy levels of an exciton in a GaAs quantum dot: Application for excitonic lasers

**DOI:** 10.1038/s41598-018-23348-9

**Published:** 2018-03-22

**Authors:** K. Luhluh Jahan, A. Boda, I. V. Shankar, Ch. Narasimha Raju, Ashok Chatterjee

**Affiliations:** School of Physics, University of Hyderabad, Hyderabad, 500046 Telangana India

## Abstract

The problem of an exciton trapped in a Gaussian quantum dot (QD) of GaAs is studied in both two and three dimensions in the presence of an external magnetic field using the Ritz variational method, the 1/*N* expansion method and the shifted 1/*N* expansion method. The ground state energy and the binding energy of the exciton are obtained as a function of the quantum dot size, confinement strength and the magnetic field and compared with those available in the literature. While the variational method gives the upper bound to the ground state energy, the 1/*N* expansion method gives the lower bound. The results obtained from the shifted 1/*N* expansion method are shown to match very well with those obtained from the exact diagonalization technique. The variation of the exciton size and the oscillator strength of the exciton are also studied as a function of the size of the quantum dot. The excited states of the exciton are computed using the shifted 1/*N* expansion method and it is suggested that a given number of stable excitonic bound states can be realized in a quantum dot by tuning the quantum dot parameters. This can open up the possibility of having quantum dot lasers using excitonic states.

## Introduction

With the advent of modern sophisticated fabrication methods such as molecular beam epitaxy, nano-lithography and etching techniques, the study of low-dimensional systems has undergone a renaissance. It is now possible to realize ultra-small semiconductor structures with quantum confinement of carriers in all the spatial directions. These structures are typically of the order of a few nanometers in size and are commonly referred to as zero-dimensional objects or more technically as quantum dots.

Interest in the subject of QDs has continued unabated for the last four decades mainly for two reasons. First and foremost, it has an intrinsic appeal because the natural length scales involved in it are of the order of a few nanometers where the quantum effects can show up in their full glory. Therefore a QD can be considered to provide a tiny laboratory where the predictions of quantum mechanics can be tested^1^. Secondly and perhaps more importantly, the QD systems exhibit very many new physical effects which are very interesting and are also quite different from those of their bulk counterparts. Furthermore, QD structures can be realized in both two and three dimensions and can also be fabricated in different sizes and shapes. This design flexibility and the novel physical effects make QD structures technologically very promising for applications in micro-electronic devices like quantum dot lasers^2^, single electron transistors^3^ and ultrafast quantum computers.

Various elementary excitations are possible in a semiconductor QD. One of the important among them is an exciton which is a bound pair of an electron and a hole. An exciton can be created by shining light on a semiconducting material. Quantum confinement can dramatically change the optical properties of a QD that depend on excitonic processes. The explanation is simple. It is well-known that the confinement per se enhances the energy of a particle in general. For an exciton, however, it increases the Coulomb attraction between the electron and the hole and thus lowers the energy. The interplay between these two contrasting effects can give rise to some interesting excitonic effects. Furthermore, the increase in the proximity between the electron and the hole due to confinement enhances the probability of radiative recombination. Therefore, one has to understand the precise effects of all these processes and also the ways to control them so that one can tune the different QD parameters to have a desired optical property.

Excitonic effects have been captured through photoluminescence experiments^4^. Excitonic effects have also been found to play a central role in optoelectronic devices such as semiconductor QDs-based photovoltaic cells and light emitting diodes^5–10^. Guo and Yu^11^ have studied the nonlinear optical susceptibilities in *Si*/*SiO*_2_ parabolic QD to determine the effect of excitons on the third harmonic generation. Their results show that the inclusion of the excitonic effect enhances the third harmonic generation by about hundred percent. Yuan *et al*.^12^ have studied the excitonic effects on the linear and non-linear optical absorptions in a parabolic QD using the exact matrix diagonalization technique and have shown that the size quantization substantially increases the optical absorption coefficients. They have furthermore shown that excitonic effects enhance the optical absorption by a factor of two.

To study the properties of a QD theoretically one has to introduce an empirical potential known as the confining potential. The simplest confining potential would of course be an infinitely deep potential well. Initial experiments^13,14^ together with the generalized Kohn theorem^15,16^, however, suggested that the confining potential in a QD would be more or less parabolic in nature. This observation has made the application of quantum mechanics to a QD system quite straightforward and consequently a large number of investigations exploring several electronic and optical properties of parabolic QDs (PQD)^17–24^ has piled up in the literature in the last few decades. Several authors have also explored the properties of excitons in PQDs^25–29^.

Some recent experiments have indicated that the confining potential in a QD is not really harmonic but rather anharmonic and has a finite depth. Recently Adamowski *et al*.^30^ have proposed a Gaussian attractive confining potential for the investigation of the properties of excess electrons in QDs. This potential has a central minimum and a finite depth and in the neighborhood of the dot centre would behave like a parabolic potential and would thus approximately satisfy the generalized Kohn theorem.

Furthermore, in contrast to the rectangular potential well, it is continuous at the dot boundary and this makes it easier to handle mathematically. Also the force experienced by the particles within this potential well is nonzero, which is again a desirable feature. The other advantages with the Gaussian confining potential vis-a-vis a parabolic potential are that the former can describe, in addition to the excitations, the ionization and tunneling processes. Masumoto and Takagahara^31^ have shown that for small QDs, the Gaussian potential is indeed a good approximation for the confining potential. The Gaussian potential has already been used by several authors as the model for confinement to study the electronic properties of a QD^32–45^. In this paper, we shall refer to a QD with a Gaussian confining potential as a Gaussian QD (GQD). Although the ground state (GS) of an exciton a GQD has been investigated by several authors, studies on the excited states of an exciton in a GQD are few and far between. The excited states of an exciton are important in both infrared spectroscopy and two-photon spectroscopy^46^. Xie^47^ has considered the exciton problem in a GQD and has studied the ground and the first excited states by using the matrix diagonalization method.

It is well known that a magnetic field provides an additional confinement and can be used to tune the confinement in a much better and cleaner way as compared to the other QD parameters which have electrostatic effects. Gu and Liang^6^ have considered the exciton problem in a GQD in the presence of a magnetic field and studied it using the matrix diagonalization method. Obviously this problem does not admit an exact solution and therefore an exact numerical solution is indeed useful. However, many a time numerical solutions fail to provide some of the interesting physics of the system. Furthermore, the wave function used in the numerical diagonalization method does not provide any insight about the state of the system in contrast to a variational method or other analytical methods. Therefore it is always desirable to develop an approximate analytical method keeping all the key features of the system into account which can give results that compare well with the numerical results. In the present work we shall calculate the GS energy and the binding energy (BE) of an exciton in a spherical GQD in the presence of an external magnetic field as a function of the QD size, magnetic field and the potential strength using the Ritz variational method, the 1/*N* expansion method and the shifted 1/*N* expansion method. We shall also investigate the correlation between the size and oscillator strength of the exciton and the QD radius. We shall also obtain all the excited states of the system for a few set of parameters. This might open up the possibility of having excitonic lasers using QDs.

## Model

The Hamiltonian of an exciton in a GQD in the presence of a magnetic field ***B*** can be written as

1 $$H=\sum _{i=e,h}[\frac{1}{2{{m}_{i}}^{\ast }}{({{\boldsymbol{p}}}_{i}+\frac{{q}_{i}}{c}{{\boldsymbol{A}}}_{i})}^{2}-{V}_{0}{e}^{-{{r}_{i}}^{2}/2{R}^{2}}]-\frac{{e}^{2}}{\varepsilon |{{\boldsymbol{r}}}_{{\boldsymbol{e}}}-{{\boldsymbol{r}}}_{h}|}\,,$$

where *i* = *e* represents an electron and *i* = *h* a hole, *q*_*e*_ denotes the electronic charge and *q*_*h*_ the hole charge, ***r***_*i*_ = ***r***_*e*_(***r***_*h*_) refers to the position vector of the electron (hole) and ***p***_*i*_ = ***p***_*e*_(***p***_*h*_) the corresponding momentum operator, ${m}_{i}^{\ast }={m}_{e}^{\ast }\,({m}_{h}^{\ast })$ is the effective mass of the electron (hole), ***A***_*i*_ = ***A***_*e*_(***A***_*h*_) measures the vector potential for the electron (hole) corresponding to the magnetic field ***B*** which has been applied in the z direction, *V*_0_ and *R* are respectively the depth and range of the single particle Gaussian confinement potential and *ε* is the dielectric constant of the material. The effective Hamiltonian can be written in the symmetric gauge (***A*** = (−*By*/2, *Bx*/2, 0)) as

2 $$H=\sum _{i=e,h\,}[-\frac{{\hslash }^{2}}{2{m}_{i}^{\ast }}{\nabla }_{i}^{2}-{V}_{0}{e}^{-{{r}_{i}}^{2}/2{R}^{2}}+\frac{1}{8}{m}_{i}^{\ast }{\omega }_{ci}^{2}{{r}_{i}}^{2}]+\frac{1}{2}({\omega }_{ce}{L}_{ze}-{\omega }_{ch}{L}_{zh})-\frac{{e}^{2}}{\varepsilon |{{\boldsymbol{r}}}_{{\boldsymbol{e}}}-{{\boldsymbol{r}}}_{{\boldsymbol{h}}}|},$$

Here we have neglected the spin of the charge carriers.

## Formulation

### Variational method

We first rewrite the Hamiltonian (2) as: *H* = *H*_0_ + *H*_1_, with

3 $${H}_{0}=\sum _{i=e,h\,}[-\frac{{\hslash }^{2}}{2{m}_{i}^{\ast }}{\nabla }_{i}^{2}+\frac{1}{2}{m}_{i}^{\ast }{\tilde{\omega }}_{i}^{2}{{\rm{r}}}_{i}^{2}]-{V}_{0}+\frac{1}{2}({\omega }_{ce}{L}_{ze}-{\omega }_{ch}{L}_{ze})-\frac{{e}^{2}}{\varepsilon |{{\boldsymbol{r}}}_{{\boldsymbol{e}}}-{{\boldsymbol{r}}}_{{\boldsymbol{h}}}|},$$

4 $${H}_{1}=-\lambda \sum _{i=e,h}[\,\frac{1}{2}{m}_{i}^{\ast }{\omega }_{i0}^{2}{r}_{i}^{2}+{V}_{0}({e}^{-\frac{{r}_{i}^{2}}{2{R}^{2}}}-1)],$$

where

5 $${\tilde{\omega }}_{i}^{2}={\omega }_{i0}^{2}+\frac{{\omega }_{ci}^{2}}{4}\,;\,{\omega }_{i0}^{2}=\frac{{V}_{0}}{{m}_{i}^{\ast }{R}^{2}},$$

and *λ* = 0 for parabolic confinement and *λ* = 1 for Gaussian confinement. We are interested in the GS of the system and therefore we drop the *L*_*z*_ − term which does not contribute to the GS energy. We assume that the sole effect of *H*_*i*_ is to renormalize the frequency ${\tilde{\omega }}_{i}$ and so we treat *H*_1_ at the mean field level. More specifically we write *H*_1_ as

6 $${H}_{1}=\lambda \sum _{i=e,h}[\frac{{V}_{0}}{\langle {{r}_{i}}^{2}\rangle }-\frac{1}{2}{m}_{i}^{\ast }{\omega }_{0i}^{2}-\frac{{V}_{0}\langle {e}^{-{{r}_{i}}^{2}/2{R}^{2}}\rangle }{\langle {{r}_{i}}^{2}\rangle }]{{r}_{i}}^{2}=\lambda \sum _{i=e,h}{\omega }_{i}^{2}{{r}_{i}}^{2},$$

where 〈${{r}_{i}}^{2}$〉 is the expectation value of ${{r}_{i}}^{2}$ with respect to the GS wave function of the harmonic oscillator of frequency ${\tilde{\omega }}_{i}$^25^. Since $\langle {{r}_{e}}^{2}\rangle =\langle {{r}_{h}}^{2}\rangle$ and $\langle {e}^{-{{r}_{e}}^{2}/2{R}^{2}}\rangle =\langle {e}^{-{{r}_{h}}^{2}/2{R}^{2}}\rangle$, we can write ${\tilde{\omega }}_{e}^{2}+2(\lambda /{m}_{e}^{\ast }){\omega }_{e}^{2}={\tilde{\omega }}_{h}^{2}+2(\lambda /{m}_{h}^{\ast }){\omega }_{h}^{2}\equiv {\omega }^{2}.$ The Gaussian potential problem thus reduces to an effective parabolic potential problem described by the Hamiltonian,

7 $$H=\frac{{{p}_{e}}^{2}}{2{m}_{e}^{\ast }}+\frac{1}{2}{m}_{e}^{\ast }{\omega }^{2}{r}_{e}^{2}+\frac{{p}_{h}^{2}}{2{{m}_{h}}^{\ast }}+\frac{1}{2}{m}_{h}^{\ast }{\omega }^{2}{r}_{h}^{2}-2{V}_{0}-\frac{2}{\varepsilon |{{\boldsymbol{r}}}_{e}-{{\boldsymbol{r}}}_{h}|}.\,$$

We now define the relative and center-of-mass (CM) coordinates ***r*** and ***R*** as: ${\boldsymbol{r}}={{\boldsymbol{r}}}_{{\boldsymbol{e}}}-{{\boldsymbol{r}}}_{{\boldsymbol{h}}}\,;\,{\boldsymbol{R}}=({m}_{e}^{\ast }{{\boldsymbol{r}}}_{e}+{m}_{h}^{\ast }{{\boldsymbol{r}}}_{h})/M$, where *M* = *m*_*e*_ + *m*_*h*_. The electron and hole momenta ***p***_***e***_ and ***p***_***h***_ can be expressed in terms of the relative momentum ***p*** = −*iħ*∇_*r*_ and the CM momentum ***P*** = −*iħ*∇_*R*_ as: ${{\boldsymbol{p}}}_{{\boldsymbol{e}}}={\boldsymbol{p}}+({m}_{e}^{\ast }/M){\boldsymbol{P}};\,{{\boldsymbol{p}}}_{{\boldsymbol{h}}}=-{\boldsymbol{p}}+({m}_{h}^{\ast }/M){\boldsymbol{P}}.$ In terms of CM and relative variables, *H* can be written as

8 $$H={H}_{R}+{H}_{r},$$

with

8a $$\begin{array}{rcl}{H}_{r} & = & -{\nabla }_{r}^{2}+\frac{1}{4}{\omega }^{2}{r}^{2}-\frac{2}{r}\,-2{V}_{0}\,,\,{H}_{R}=-{\nabla }_{R}^{2}+\frac{1}{4}{\omega }^{2}{R}^{2},\\ \omega  & = & {[(1-\lambda )\frac{{\omega }_{0}^{2}}{2}+\frac{{\omega }_{c}^{2}}{8}+\frac{2\tilde{\omega }\lambda {V}_{0}}{3}-\frac{2\tilde{\omega }\lambda {V}_{0}}{3}{(\frac{\tilde{\omega }{R}^{2}}{1+\tilde{\omega }{R}^{2}})}^{3/2}]}^{1/2}.\end{array}$$

where all energies are measured in units of Rydberg ${R}_{y}^{\ast }=(\mu {e}^{4}/2{\hslash }^{2}{\varepsilon }^{2})$ and the lengths in units of the Bohr radius ${a}_{0}^{\ast }=({\hslash }^{2}\varepsilon /\mu {e}^{2})$, $\mu (=[{m}_{e}^{\ast }{m}_{h}^{\ast }]/M)$ being the reduced mass.

If ${\rm{\Psi }}({{\boldsymbol{r}}}_{{\boldsymbol{e}}},{{\boldsymbol{r}}}_{{\boldsymbol{h}}})$ is the eigenfunction of *H*, then we can write: ${\rm{\Psi }}({{\boldsymbol{r}}}_{{\boldsymbol{e}}},{{\boldsymbol{r}}}_{{\boldsymbol{h}}})={\rm{\varphi }}({\boldsymbol{r}})\chi ({\boldsymbol{R}})$, ${\rm{\varphi }}({\boldsymbol{r}})$ and *χ*(***R***) being the eigenfunctions of *H*_*r*_ and *H*_*R*_ belonging to the eigenvalues *E*_*r*_ and *E*_*R*_ respectively. The exciton energy *E* is then given by: *E* = *E*_*R*_ + *E*_*r*_, where *E*_*R*_(=3*ω*/2) is the GS energy (GSE) for the CM motion in three dimensions. The exact analytical evaluation of *E*_*r*_ is not possible in general. So we use the Ritz variational method with the trial function: ${\rm{\varphi }}({\boldsymbol{r}}) \sim {e}^{-\alpha {r}^{2}-\beta r}$, where *α* and *β* are variational parameters. The exciton binding energy (BE) *E*_*b*_ is defined as: *E*_*b*_ = (*E*_*e*_ + *E*_*h*_ − *E*), where *E*_*e*_ and *E*_*h*_ are respectively the electron and hole GSE in the same QD. The exciton size is given by: $\langle r\rangle =\langle {\rm{\Psi }}|r|{\rm{\Psi }}\rangle$.

The exciton oscillator strength is another important quantity of interest. In the envelop-function approximation, the exciton oscillator strength^26^ can be written as

9 $$\,{f}_{ex}=\frac{2{P}^{2}}{{m}_{0}({E}_{ex}-{E}_{0})}{|{\int }^{}{\rm{\Psi }}({{\boldsymbol{r}}}_{{\boldsymbol{e}}},{{\boldsymbol{r}}}_{{\boldsymbol{e}}})d{r}_{e}|}^{2},$$

where *P* describes intracell matrix-element effects, *m*_0_ is the bare electron mass, *E*_*ex*_ − *E*_0_ = *E* + *E*_*g*_, *E*_*g*_ being the optical band gap. From the definition of centre of mass and relative coordinates and the definition ${\rm{\Psi }}({{\boldsymbol{r}}}_{{\boldsymbol{e}}},{{\boldsymbol{r}}}_{{\boldsymbol{h}}})=\chi ({\boldsymbol{R}}){\rm{\varphi }}({\boldsymbol{r}})$, we have

10 $${\rm{\Psi }}({{\boldsymbol{r}}}_{{\boldsymbol{e}}},{{\boldsymbol{r}}}_{{\boldsymbol{e}}})=\chi ({{\boldsymbol{r}}}_{{\boldsymbol{e}}})\varphi (0),$$

so that (9) reduces to

11 $${f}_{ex}=\frac{2{P}^{2}}{{m}_{0}({E}_{ex}-{E}_{0})}{|{\rm{\varphi }}(0)|}^{2}{|{\int }^{}\chi ({r}_{{\boldsymbol{e}}})d{r}_{e}|}^{2}.$$

### 1/*N* expansion method

The 1/*N* expansion method, where *N* is the number of spatial dimensions, is a very useful technique for the calculation of the eigenvalues for spherically symmetric potentials^48–51^. The 1/*N* expansion method uses 1/*k* = 1/(*N* + 2*l*) as the expansion parameter, where *l* is the angular momentum quantum number and therefore it is often referred to as a non-perturbative technique and is valid for the entire range of the coupling parameters. The 1/*N* expansion method is, however, at times plagued with slow convergence, particularly for higher excited states. To avoid this difficulty, Sukhatme and Imbo^47^ have introduced a shifted 1/*N* expansion method which brings in an extra degree of freedom ‘*a*’ in the expansion parameter which is now given by 1/*k* = 1/(*N* + 2*l* − *a*).

The radial part *R*(*r*) of the *N*-dimensional (*ND*) Schr$\ddot{o}$dinger equation for a spherically symmetric potential *V*(*r*) is given by

12 $$[-\frac{{\hslash }^{2}}{2\mu }(\frac{{d}^{2}}{d{r}^{2}}+\frac{N-1}{r}\frac{d}{dr})+\frac{l(l+N-2)}{2{r}^{2}}+V(r)]R=ER,$$

which on substituting *R*(*r*) = *r*^− (*N*−1)/2^*u*(*r*), reduces to the effective one-dimensional equation

13 $$-\frac{{\hslash }^{2}}{2\mu }\frac{{d}^{2}u}{d{r}^{2}}+(\frac{(k-1)(k-3)}{8\mu {r}^{2}}+{k}^{2}\tilde{V}(r))u=Eu\,,$$

where *k* = (*N* + 2*l*) and $\tilde{V}(r)=V(r)/{k}^{2}$. Eq. (13) is the starting point in the unshifted 1/*N* expansion, whereas in the shifted 1/*N* expansion one introduces an additional parameter ‘*a*’ in terms of which Eq. (13) can be written as

14 $$-\frac{{d}^{2}u}{d{r}^{2}}+(\frac{[\bar{k}-(1-a)][\bar{k}-(3-a)]}{4{r}^{2}}+{\bar{k}}^{2}\tilde{V}(r))u=Eu,$$

where $\bar{k}=(N+2l-a)$, $\tilde{V}(r)=V(r)/{\bar{k}}^{2}$ and the units have been chosen so that *ħ* = 2*μ* = 1. In the limit of large $\bar{k}\,(N\to \infty ),$ the energy eigenvalue to leading order in $\bar{k}\,$is given by

15 $${E}_{\infty }={\bar{k}}^{2}(1/4{r}_{0}^{2}+\tilde{V}({r}_{0})),$$

where *r*_0_ is to be determined by minimizing the effective potential ${V}_{eff}=(1/4{r}_{0}^{2}+\tilde{V}({r}_{0}))$. We now define: $x=-{\bar{k}}^{1/2}(1-r/{r}_{0})$ and expand (15) in a Taylor series around *x* = 0 to get

16 $$(-\,\frac{{d}^{2}}{d{x}^{2}}\,+\,\frac{{{\rm{\Omega }}}^{2}}{4}{x}^{2}+{\varepsilon }_{0}+\hat{V}(x))u(x)=\lambda u(x),$$

where

17 $${\rm{\Omega }}={(3+\frac{{r}_{0}V\text{'}\text{'}({r}_{0})}{V\text{'}({r}_{0})})}^{1/2},\,{\varepsilon }_{0}=\frac{\bar{k}}{4}-\frac{(2-a)}{2}+\frac{(1-a)(3-a)}{4\bar{k}}+{{r}_{0}}^{2}\bar{k}\tilde{V}({r}_{0})\,,\,\lambda =\frac{E{r}_{0}^{2}}{\bar{k}\,},$$

and

18 $$\begin{array}{rcl}\hat{V}(x) & = & \frac{1}{{\bar{k}}^{1/2}}({\varepsilon }_{1}x+{\varepsilon }_{3}{x}^{3})+\frac{1}{\bar{k}}({\varepsilon }_{2}{x}^{2}+{\varepsilon }_{4}{x}^{4})+\frac{1}{{\bar{k}}^{3/2}}\\  &  & \times ({\delta }_{1}x+{\delta }_{3}{x}^{3}+{\delta }_{5}{x}^{5}+\frac{1}{{\bar{k}}^{2}}({\delta }_{2}{x}^{2}+{\delta }_{4}{x}^{4}+{\delta }_{6}{x}^{6})+\cdots ),\end{array}$$

with

19 $$\begin{array}{rcl}{\varepsilon }_{1} & = & (2-a),\\ {\varepsilon }_{2} & = & -\frac{3(2-a)}{2},\\ {\varepsilon }_{3} & = & [-1+\frac{{r}_{0}^{5}{V}^{{{\prime}\,{\prime\prime}} ({r}_{0})}}{6{\bar{k}}^{2}}],\\ {\varepsilon }_{4} & = & [\frac{5}{4}+{r}_{0}^{6}{V}^{{{\prime\prime}\,{\prime\prime}} ({r}_{0})}/24{\bar{k}}^{2}],\\ {\delta }_{1} & = & -\frac{(1-a)\,(3-a)}{2},\\ {\delta }_{2} & = & \frac{3(1-a)\,(3-a)}{4},\\ {\delta }_{3} & = & 2(2-a),\\ {\delta }_{4} & = & -\frac{5(2-a)}{2},\\ {\delta }_{5} & = & -\frac{3}{2}+\frac{{r}_{0}^{7}{V}^{{{\prime}\,{\prime\prime} \,{\prime\prime}} ({r}_{0})}}{120{\bar{k}}^{2}},\\ {\delta }_{6} & = & \frac{7}{4}+\frac{{r}_{0}^{8}{V}^{{{\prime\prime} \,{\prime\prime}\,{\prime\prime}} ({r}_{0})}}{720{\bar{k}}^{2}}.\end{array}$$

Applying the fourth-order Rayleigh-Schr$\ddot{o}$dinger perturbation theory to the perturbed Harmonic oscillator (16), we obtain

20 $${\lambda }_{n}={\lambda }_{n}^{(0)}+{\lambda }_{n}^{(1)}+{\lambda }_{n}^{(2)}+{\lambda }_{n}^{(3)}+{\lambda }_{n}^{(4)}+\cdots $$

21a $${\lambda }_{n}^{(0)}={\varepsilon }_{0}+(n+\frac{1}{2}){\rm{\Omega }}$$

21b $$\begin{array}{rcl}{\lambda }_{n}^{(1)} & = & g[(1+2n){\tilde{\varepsilon }}_{2}+3(1+2n+2{n}^{2}){\tilde{\varepsilon }}_{4}]+{g}^{2}[(1+2n){\tilde{\delta }}_{2}\\  &  & +\,3(1+2n+2{n}^{2}){\tilde{\delta }}_{4}+5(3+8n+6{n}^{2}+4{n}^{3}){\tilde{\delta }}_{6}]\end{array}$$

21c $$\begin{array}{rcl}{\lambda }_{n}^{(2)} & = & -\frac{g}{{\rm{\Omega }}}[{\tilde{\varepsilon }}_{1}^{2}+6(1+2n){\tilde{\varepsilon }}_{1}{\tilde{\varepsilon }}_{3}+(11+30n+30{n}^{2}){\tilde{\varepsilon }}_{3}^{2}]\\  &  & -\,\frac{{g}^{2}}{{\rm{\Omega }}}[(1+2n){\tilde{\varepsilon }}_{2}^{2}+12(1+2n+2{n}^{2}){\tilde{\varepsilon }}_{2}{\tilde{\varepsilon }}_{4}\,\\  &  & +\,2(21+59n+51{n}^{2}+34{n}^{3}){\tilde{\varepsilon }}_{4}^{2}+2{\tilde{\varepsilon }}_{1}{\tilde{\delta }}_{1}]\,+O({g}^{3})\end{array}$$

21d $$\begin{array}{rcl}{\lambda }_{n}^{(3)} & = & \frac{{g}^{2}}{{{\rm{\Omega }}}^{2}}[4{\tilde{\varepsilon }}_{1}^{2}{\tilde{\varepsilon }}_{2}+36(1+2n){\tilde{\varepsilon }}_{1}{\tilde{\varepsilon }}_{2}{\tilde{\varepsilon }}_{3}+24(1+2n){\tilde{\varepsilon }}_{1}^{2}{\tilde{\varepsilon }}_{4}\\  &  & +\,8(11+30n+30{n}^{2}){\tilde{\varepsilon }}_{2}{\tilde{\varepsilon }}_{3}^{2}+8(31+78n+78{n}^{2}){\tilde{\varepsilon }}_{1}{\tilde{\varepsilon }}_{3}{\tilde{\varepsilon }}_{4}\\  &  & +\,12(57+189n+225{n}^{2}+150{n}^{3}){\tilde{\varepsilon }}_{3}^{2}{\tilde{\varepsilon }}_{4}]+O({g}^{3})\end{array}$$

21e $$\begin{array}{rcl}{\lambda }_{n}^{(4)} & = & -\frac{{g}^{2}}{{{\rm{\Omega }}}^{3}}[8{\tilde{\varepsilon }}_{1}^{3}{\tilde{\varepsilon }}_{3}+108(1+2n){\tilde{\varepsilon }}_{1}^{2}{\tilde{\varepsilon }}_{3}^{2}+48(11+30n+30{n}^{2}){\tilde{\varepsilon }}_{1}{\tilde{\varepsilon }}_{3}^{3}\\  &  & +\,30(31+109n+141{n}^{2}+94{n}^{3}){\tilde{\varepsilon }}_{3}^{4}]+O({g}^{3}),\end{array}$$

where $g=1/\bar{k}$, ${\tilde{\varepsilon }}_{j}={\varepsilon }_{j}/{(2\mu \Omega /\hslash )}^{j/2}$, ${\mathop{\delta }\limits^{ \sim }}_{{\rm{j}}}={\delta }_{{\rm{j}}}/{(2\mu {\rm{\Omega }}/\hslash )}^{j/2}.$ In the usual 1/*N* expansion method *a* = 0, while in the shifted method, ‘*a*’ is to be determined from the condition *E*^(−1)^ = 0, which gives *a* = 2 − (2*n* + 1)Ω. In our problem *V*(*r*) = *ω*^2^*r*^2^/4 − 2/*r* − 2*V*_0_. We have calculated energies using both the unshifted and the shifted 1/*N* expansion methods.

## GS Results and Discussion

**Figure 1:**
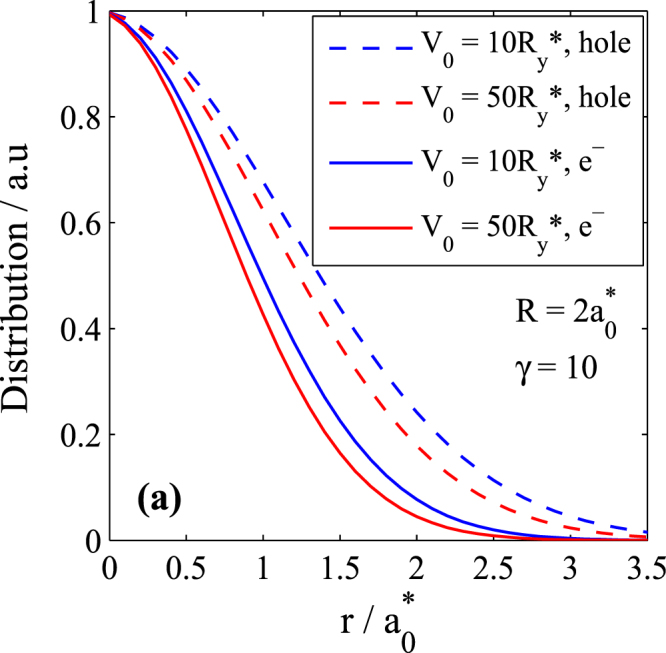
Distribution of electron and hole of an exciton in a GaAs QD for two values of the potential depth *V*_0_.

The methods discussed above are quite general in nature and can be applied to any quantum dot. But, for the sake of concreteness, we apply them to a GaAs QD. So, for the material parameters we take: *ε* = 12.8, ${m}_{e}^{\ast }=0.067\,{m}_{e}$ and ${m}_{h}^{\ast }=0.099\,{m}_{e}$ (light-hole mass). Thus we have ${a}_{0}^{\ast }=17.7\,\,nm$, ${R}_{y}^{\ast }=3.1\,\,meV$, *E*_*g*_ = 1.51 *eV*, *P*^2^/*m*_0_ = 1 *eV*. We also define for the sake of convenience a parameter $\gamma =e\hslash B/2\mu c{R}_{y}^{\ast }$ which is proportional to the magnetic field strength. One can see that 1*γ* = 0.47226*B*(*T*)^22,26^. Figure 1 shows the distribution of the electron and hole of an exciton in a GaAs QD. One can see from the figure that as the potential depth increases, the distribution decreases. The distribution of the hole is little larger than that of the electron. We also find that, as the magnetic field increases, the distribution decreases (not shown here). We furthermore find that while the parabolic model underestimates the electron distribution, it overestimates the hole distribution (not shown here).

**Figure 2:**
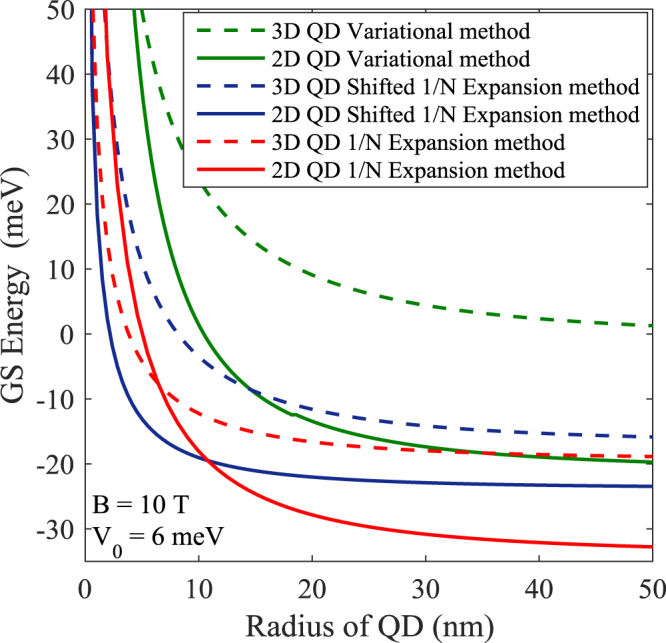
Exciton GSE as a function of QD radius in 3D and 2D Gaussian GaAs QD in a magnetic field of 10 Tesla.

In Fig. 2 we show the variation of the exciton GSE of a light-hole exciton in a 3D GaAs QD as a function of the effective QD size (*R*) for potential depth of *V*_0_ = 6 *meV* and magnetic field of *B* = 10 *T*. We have plotted results obtained from the 1/*N* expansion method, shifted 1/*N* expansion method and the variational method for both 2D and 3D confinement. In all the three cases as *R* decreases, the exciton energy monotonically increases and this increase becomes very rapid below a certain critical value of *R*. The reason is understandable. As *R* decreases, the uncertainty in the exciton momentum increases leading to an increase in the kinetic energy of the exciton. Thus as *R* decreases, GSE increases in general. Also we can observe that the GSE is lower in 2D than in 3D because the confinement is stronger in 2D. While the variational method gives the upper bound to GSE, the 1/*N* expansion gives a lower bound. The shifted-1/*N* method is found to give GSE that lies between those given by the other two methods.

**Figure 3:**
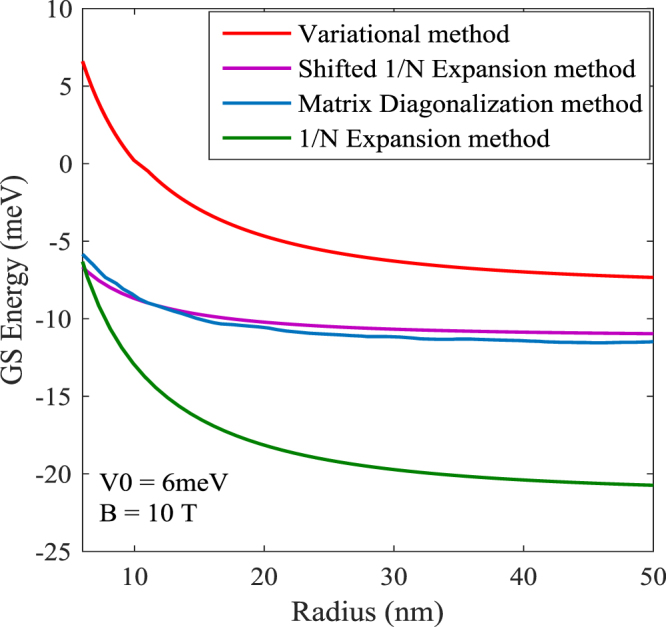
Comparison of our results for the exciton GSE with those from Matrix diagonalization method of Gu and Liang.

In Fig. 3 we have compared the GSE results determined from the aforementioned methods with those obtained from the matrix diagonalization method by Gu and Liang^6^. It is clearly evident that the shifted-1/*N* results are in very good agreement with the exact numerical results.

**Figure 4:**
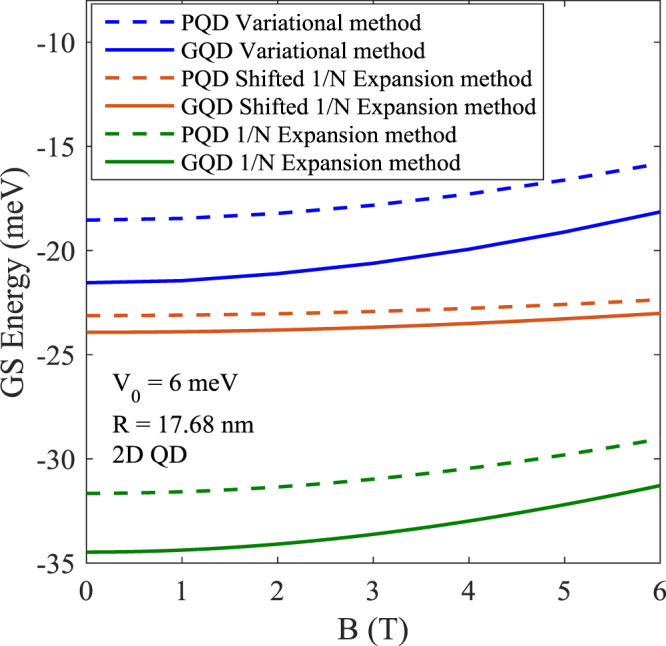
Exciton GSE as a function of *B* in 2D PQD and GQD of GaAs.

Figure 4 shows the variation of the exciton GSE as a function of the magnetic field *B* for 2D QD with *V*_0_ = 6 *meV* and *R* = 17.68 *nm*. As *B* increases, GSE also increases which is again on the expected line. Though the qualitative behavior of the exciton GSE is same in PQD and GQD, the parabolic confinement seems to overestimate the energy. Also the nature of confinement becomes unimportant when the QD size becomes large. We would like to mention here that the shifted 1/*N* expansion method not only provides results that are in excellent agreement with the exact matrix diagonalization method, but it also has a few distinct advantages over the exact diagonalization method. First, as we have already alluded to in the introduction, it provides an analytical expression for the energy which is always preferable. Secondly, it allows us to calculate the wave functions of the system analytically and can thus provide a much better understanding of the physics of the system. Thirdly, once the wave functions are obtained, one can calculate average values of several dynamical variables. Finally, since this method provides the entire energy spectrum, one can also calculate all the important thermodynamic quantities.

**Figure 5:**
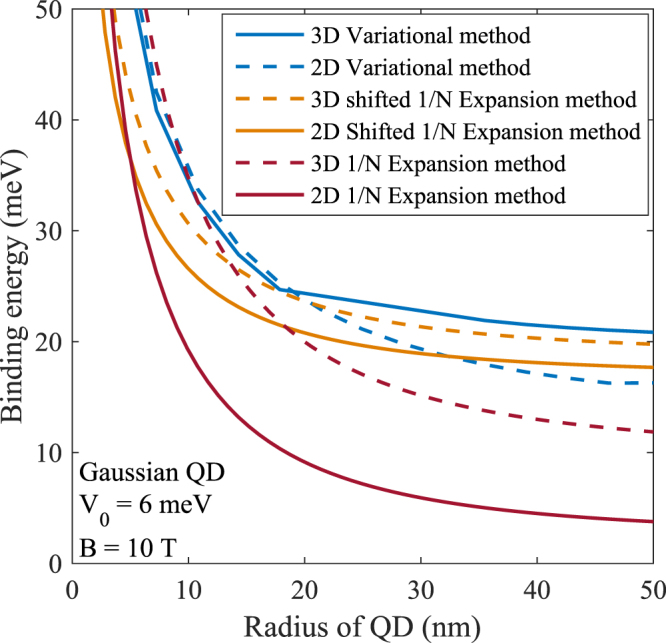
BE vs *R* for 2D and 3D Gaussian GaAs QDs for *B* = 10 *T*.

**Figure 6:**
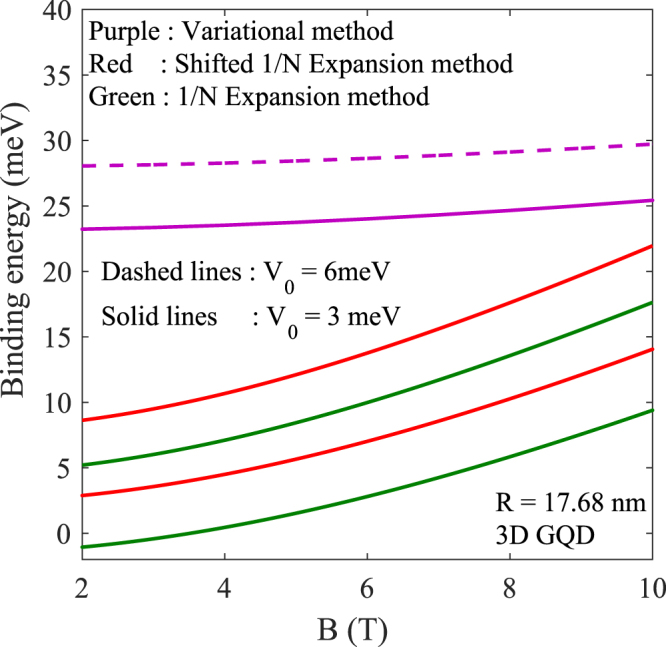
Exciton BE energy as a function of *B* in a GQD for two values of *V*_0_.

The variation of exciton BE of a GQD is shown in Fig. 5. It may be noted that since the confining potential is negative for all finite values of the electron and hole coordinates, one would expect that bound state of an exciton should correspond to a negative energy value. However for this bound state to be stable it is necessary that the BE energy as defined earlier should be positive. We see that as the QD size decreases, BE increases and the rate of increase is larger for smaller dots. This is understandable because with a decrease in the dot size, the spatial overlap between an electron and a hole increases leading to a stronger coulomb binding. Comparison of results for 2D and 3D QDs suggests that the BE for a QD is larger in 2D than in 3D. As suggested by Fig. 3, the BE results obtained from the shifted 1/*N* expansion should be trusted the most. In Fig. 6 we plot the behavior of the exciton BE as a function of the magnetic field for two values of the potential depth. As expected, BE increases with increasing magnetic field. We find that BE is much larger in a GQD than in a PQD.

**Figure 7:**
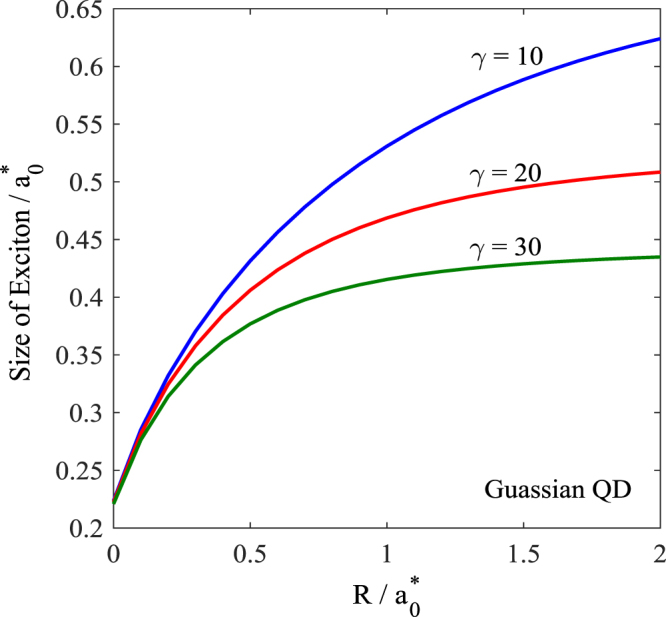
Light-hole exciton size in a 3D GQD as a function of the QD size.

**Figure 8:**
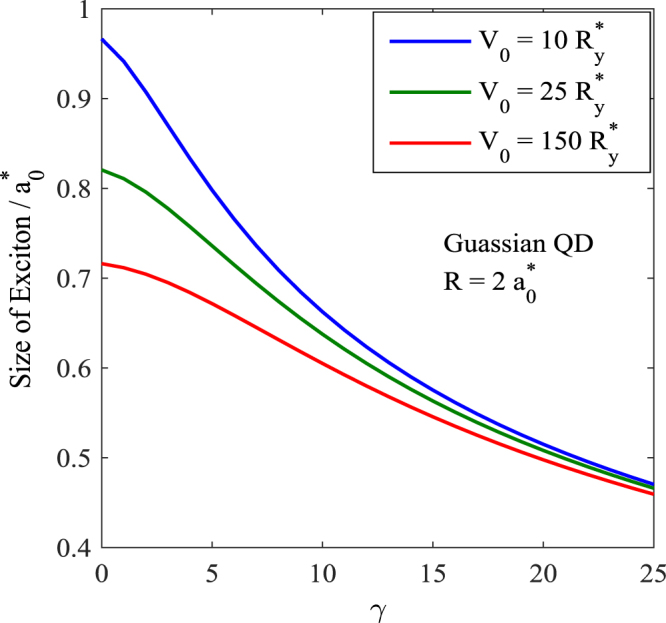
Variation of the size of the light-hole exciton in a 3D GQD as a function of the magnetic field.

In Fig. 7 we plot the variation of the exciton size as a function of *R*. The figure shows that as *R* increases, the size of the exciton also increases. However, the exciton size seems to saturate as *R* increases beyond some critical value which may be identified as the bulk limit. We have plotted the exciton size vs. *R* for three different values of the magnetic field for both PQD and GQD. As the magnetic field increases, the size of an exciton decreases. This is a direct consequence of the localizing property of the magnetic field. Figure 8 gives the variation of the exciton size as a function of the magnetic field. As expected, the exciton size decreases with increasing magnetic field. Also the exciton size decreases as the potential becomes deeper.

One can make another observation from the Figs 5 and 7. According to Fig. 7, the size of the exciton increases as the QD size increases, while according to Fig. 5, the binding energy decreases with increasing QD size. Thus one can conclude that as the size of the exciton increases, the binding energy decreases.

**Figure 9:**
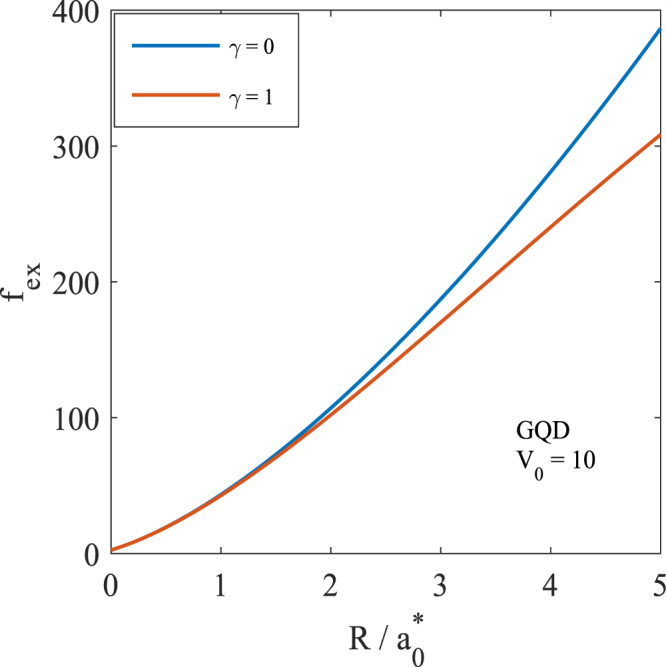
GS oscillator strength of an exciton in a GQD for two different values of the magnetic field.

We have also studied the variation of the exciton oscillator strength *f*_*ex*_ as a function of *R* and results are shown in Fig. 9. One can see that as *R* decreases, *f*_*ex*_ also decreases. This happens because as *R* decreases, the exciton energy increases and hence *f*_*ex*_ decreases. Figure 9 also shows that *f*_*ex*_ decreases in the presence of a magnetic field. Again the explanation is simple. The application of the magnetic field leads to an additional confinement, which induces an enhancement in the exciton energy and hence the oscillator strength decreases in the presence of a magnetic field.

### Excited states

From Eqs (20) and (21) we can calculate the energy of all the excited states. Table 1 shows the results for the energy spectrum of a Gaussian GaAs QD with radius *R* = 88.5 *nm* and potential depth *V*_0_ = 12 *meV* placed in a magnetic field *B* = 1 *T*. One can easily see that this QD can sustain a finite number of bound states, namely 18 bound states. However it should be pointed out that one has to calculate the BE of these states to make sure whether these are stable bound states. Here we shall loosely call them bound states. On the other hand, if the exciton energy comes out to be negative for a state, then one can state with certainty that it does not qualify to be a bound state. However it can be a scattering state. Though it is true that the energy spectra in a QD should be discrete, the Rydberg states are expected to be much less discrete because of the form of the potential at the boundary where the potential smoothly goes to zero. This makes the tunneling-like processes possible. By tuning the QD parameters, one can perfectly control the number of bound states. For example, with, *R* = 53 *nm*, *V*_0_ = 3.98 *meV* and *B* = 4 *T*, one obtains at most four bound states as shown in Table 2. In Table 3 we explicitly show the binding energy values for a QD with certain parameters.

**Table 1:** The energy spectra of an exciton in a 2D Gaussian GQD calculated by shifted 1/*N* expansion method with *R* = 88.5 *nm*, *V*_0_ = 12 *meV* and *B* = 1*T*. Energies are expressed in meV.

*l*	0	1	2	3	4	5	6	7	8	9	10
n											
0	−27.46	−15.88	−15.36	−15.08	−14.85	−14.63	−14.43	−14.24	−14.05	−13.86	9.698
1	−16.25	−15.23	−13.09	−8.57							
2	−14.19	−9.59	−1.47	11.38							
3	−5.86	5.97									
4	14.17

**Table 2:** Energy levels of an exciton in a Gaussian GaAs QD with *R* = 53 *nm*, *V*_0_ = 3.98 *meV* and *B* = 4 *T*.

*l*	0	1	2	3
n				
**0**	−17.39	−5.088	−3.732	15.18
**1**	−1.603	28.43		
**2**	60.274

**Table 3:** Binding energy of an exciton in a Gaussian QD with *R* = 17.7 *nm*,*V*_0_ = 3.98 *meV* and *B* = 1 *T*.

*l*	0	1	2	3	4	5	6	7	8	9	10
n											
0	12.68	10.46	9.52	8.23	6.53	4.42	3.25	2.15	1.28	0.32	0.032
1	9.23	6.79	5.42	3.67	1.51	0.73					
2	8.11	5.43	4.67	2.77	0.76						
3	3.78	2.63	1.97	1.28	0.52						
4	1.52										

**Figure 10:**
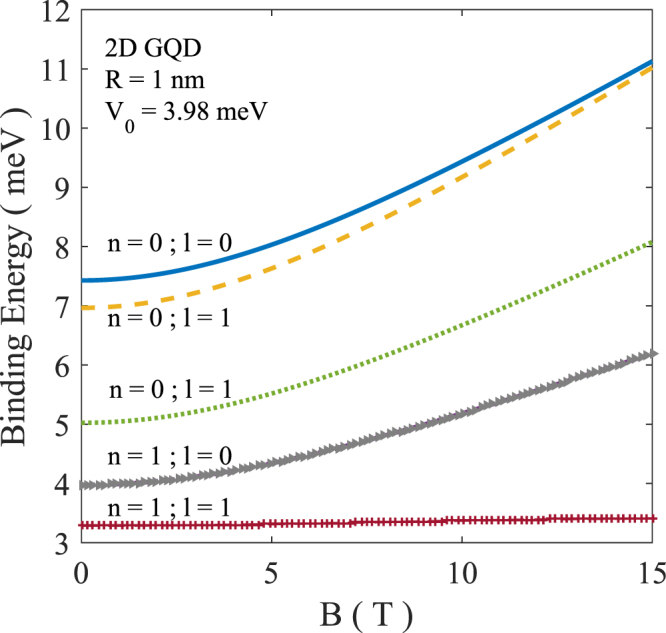
BE of a few excitonic states as a function of the magnetic field.

We have also studied the behavior of the BE of an exciton in different states as a function of the magnetic field. Results are plotted in Fig. 10. It is clear that BE is smaller for a higher excited state. On the other hand, BE increases with increasing magnetic field which is expected in view of the additional confinement provided by the magnetic field.

## Conclusion

In the present paper, we have calculated the GS energy, binding energy and the size of an exciton in a Gaussian GaAs QD in two and three dimensions in the presence of an external magnetic field using the Ritz variational method, 1/*N* expansion method and shifted-1/*N* expansion method. While the variational method gives the upper bound to the GS energy, 1/*N* method gives the lower bound. We have also compared our results with those for the corresponding parabolic model. It turns out that the electron/hole distribution decreases as the confining potential becomes deeper and also as the magnetic field becomes stronger.

We have shown that the exciton energy decreases with increasing potential depth. With decreasing QD size, the exciton energy increases and becomes significantly large below a certain critical size. The shifted 1/*N* expansion results agree excellently well with those obtained from the exact numerical diagonalization method.

We have furthermore shown that the exciton binding energy increases with decreasing dot size and it is underestimated by the parabolic potential model. Also the binding becomes stronger as the potential becomes deeper and the magnetic field is increased. It is shown that the exciton size decreases as the dot becomes smaller or the potential becomes deeper or the magnetic field is increased. Next we have shown that the GS exciton oscillator strength decreases as the QD size decreases or the external magnetic field increases. We have also calculated the excited state energies of an exciton in a 2D GQD in the presence of a magnetic field. It is observed that by tuning the QD parameters one can have a given number of bound states in a QD by suitably tuning the QD parameters. In this context, we have also explicitly shown an example where one can have at most four bound states in a Gaussian GaAs QD. This tunability may have potential applications in QD lasers. In this work we have however neglected the sin-Zeeman term. The interplay between the electron-hole interaction and the magnetic field effect through the spin-Zeeman term may have some important consequences which will be taken up for investigation in a subsequent study.
